# Prenatal Antidepressant Exposure and Risk of Spontaneous Abortion – A Population-Based Study

**DOI:** 10.1371/journal.pone.0072095

**Published:** 2013-08-28

**Authors:** Maiken Ina Siegismund Kjaersgaard, Erik Thorlund Parner, Mogens Vestergaard, Merete Juul Sørensen, Jørn Olsen, Jakob Christensen, Bodil Hammer Bech, Lars Henning Pedersen

**Affiliations:** 1 Department of Public Health, Section for Biostatistics, Aarhus University, Aarhus, Denmark; 2 Research Unit for General Practice, Aarhus University, Aarhus, Denmark; 3 Department of Public Health, Section for General Practice, Aarhus University, Aarhus, Denmark; 4 Regional Center for Child and Adolescent Psychiatry, Aarhus University Hospital, Risskov, Denmark; 5 Department of Public Health, Section for Epidemiology, Aarhus University, Aarhus, Denmark; 6 Department of Neurology, Aarhus University Hospital, Aarhus, Denmark; 7 Department of Clinical Pharmacology, Aarhus University, Aarhus, Denmark; 8 Department of Obstetrics and Gynecology, Institute of Clinical Medicine, Aarhus University, Aarhus, Denmark; University of Tennessee Health Science Center, United States of America

## Abstract

**Purpose:**

To estimate the risk of spontaneous abortion after use of antidepressant medication during pregnancy.

**Methods:**

From the Danish Medical Birth Registry and the Danish National Hospital Registry, we identified all pregnancies leading to in- or outpatient contacts in Denmark from February 1997 to December 2008. The Danish Registry of Medicinal Product Statistics provided information on the women's prescriptions for antidepressants during pregnancy. We obtained information on women who were diagnosed with depression from the Danish Psychiatric Central Registry. Adjusted relative risks (aRR) of spontaneous abortion were estimated according to exposure to antidepressants or maternal depression using binomial regression.

**Results:**

Of the 1,005,319 pregnancies (547,300 women) identified, 114,721 (11.4%) ended in a spontaneous abortion. We identified 22,061 pregnancies exposed to antidepressants and 1,843 with a diagnosis of depression with no antidepressant use, of which 2,637 (12.0%) and 205 (11.1%) ended in a spontaneous abortion, respectively. Antidepressant exposure was associated with an aRR of 1.14 (95% confidence interval (CI) 1.10–1.18) for spontaneous abortion compared with no exposure to antidepressants. Among women with a diagnosis of depression, the aRR for spontaneous abortion after any antidepressant exposure was 1.00 (95% CI 0.80–1.24). No individual selective serotonin reuptake inhibitor (SSRI) was associated with spontaneous abortions. In unadjusted analyses, we found that mirtazapine, venlafaxine, and duloxetine were associated with spontaneous abortions among women with depression but we had no information on potential differences in disease severity and only few pregnancies were exposed in the population.

**Conclusion:**

We identified a slightly increased risk of spontaneous abortion associated with the use of antidepressants during pregnancy. However, among women with a diagnosis of depression, antidepressants in general or individual SSRI in particular were not associated with spontaneous abortions. Further studies are warranted on the newer non-SSRI antidepressants, as we had insufficient data to adjust for important confounding factors.

## Introduction

Depression is a common condition during pregnancy affecting 7–14% of all women [1], [2]. Some women have depressive symptoms when they enter pregnancy while others develop symptoms during pregnancy [1], [2]. These women and their health care providers are faced with a treatment decision where they have to balance the potential benefits and risks of both disease and treatment.

Spontaneous abortion affects 10–15% of clinically recognized pregnancies and has been linked to both maternal depression [3] and antidepressant use during pregnancy [3]–[5]. However, existing studies on antidepressant use during pregnancy and risk of abortion have been limited by e.g. low power [6]. The largest study published found that paroxetine and venlafaxine were associated with spontaneous abortions but the results were based on only 84 children exposed to paroxetine and 33 children exposed to venlafaxine [4]. For some types of antidepressants, e.g. duloxetine, there is no published data on the potential risk of spontaneous abortion.

Lack of sufficiently powered studies is a clinical problem in preconception and early pregnancy when counseling women with depression. We therefore aimed to evaluate the risk of spontaneous abortion among women exposed to any antidepressant and to specific types of antidepressants in a large population-based cohort of pregnant women, and further attempted to adjust for confounding by indication by making internal comparisons among women with a diagnosis of depression in the registries.

## Methods

We included all clinically recognized pregnancies in Denmark with an estimated conception and an observed pregnancy outcome in the period 1 February 1997 to 31 December 2008. Information was obtained from the Danish administrative health registries and linked through the CPR-number, a unique identification number given to all citizens. We specifically investigated in- or outpatient contacts with a diagnosis of spontaneous abortion before 22 weeks of gestation (in Denmark, a child born after 22 weeks of gestation is considered as either stillborn or live born), but also included information on pregnancies that ended with a molar pregnancy, ectopic pregnancy, induced abortion, stillbirth or live birth.

### Pregnancy outcomes

Clinically recognized abortions were identified in the Danish National Hospital Registry. This registry contains data on in- and outpatient contacts in Denmark coded according to a Danish version of the 10th revision of the International Classification of Diseases (ICD-10) since 1995 [7]. Abortions (ICD-10: O02.0–O06.9) were divided in spontaneous abortions (ICD-10: O02.0–O03.9), induced abortions (ICD-10: O04.0–O05.2, O05.5–O06.9), or induced abortions due to fetal disease (ICD-10: O05.3, O05.4). Molar or ectopic pregnancies (ICD-10: O00.0–O01.9) were excluded from the main analyses. Furthermore, diagnoses regarding failed induced abortions (ICD-10: O07) were disregarded as we assume that a failed induced abortion was followed by an abortion, a stillbirth or a live birth, which were all diagnosed separately. Live births and stillbirths were identified in the Danish Medical Birth Registry, which holds information on all births in Denmark [8].

The pregnancy period was defined from the estimated date of conception to the date of the outcome (induced or spontaneous abortion, stillbirth or live birth). For abortions (spontaneous or induced), the gestational age was based on the Danish National Hospital Registry, and for the live births and stillbirths it was based on the Medical Birth Registry. Gestational age before 12 weeks was normally based on last menstrual period and on ultrasound scans for later gestations when available. Missing gestational age for abortions was replaced by the median of non-missing values of the gestational age for abortions (N = 7,125; 2.5% of all abortions).

We performed a hierarchical coding of the pregnancies to take repeated contacts into consideration. Any stillbirth or live birth resulted in recoding of other prior endpoints in the index pregnancy period. Pregnancies with multiple codes for abortion were coded as a spontaneous abortion if the pattern of codes indicated such, even if the index period also included codes for induced abortion (e.g. in case of a spontaneous abortion in the time period between the initial contact and the planned surgical termination). Pregnancies including codes for induced abortions due to fetal disease (ICD-10: Q05.3,Q05.4), were coded as such, and the remaining pregnancies with codes for abortion were coded as induced abortion not due to fetal disease.

### Antidepressant exposure

We obtained information on all redeemed prescriptions in Denmark from the Registry of Medicinal Product Statistics. We included information on all redeemed prescriptions from 1 January 1996 to 31 December 2008. Antidepressants were defined according to the Anatomical Therapeutic Chemical (ATC) code N06A, and we specifically assessed the prescriptions of selective serotonin reuptake inhibitors (SSRI), ATC code: N06AB, and tricyclic antidepressants (TCA), ATC code: N06AA, and ‘other’ (serotonin–norepinephrine reuptake inhibitors (SNRI), ATC codes: N06AX16 and N06AX21; noradrenergic and specific serotonergic antidepressants (NaSSA), ATC codes: N06AX03 and N06AX11; and norepinephrine-dopamine reuptake inhibitors, ATC code: N06AX12). Pregnancies were considered “exposed” to antidepressants if the mother had redeemed a prescription for antidepressant medication at any time from 30 days before conception up to 1 day before the end of pregnancy. Pregnancies were classified as “unexposed” if the mother had not redeemed any prescription for antidepressant medication from 6 months before conception up to 1 day before the end of the pregnancy. Thus, in an attempt to avoid misclassification, we excluded from the analyses pregnancies in which the women redeemed a prescription for an antidepressant anytime from 6 months to 30 days prior to conception but without redeeming any prescription for an antidepressant in the index pregnancy period from 30 days before conception up to 1 day before the end of pregnancy (N = 11,834 pregnancy outcomes). In sensitivity analyses, we included all these women as either exposed or unexposed, respectively. In further sensitivity analyses, we considered unexposed to be women who did not redeem a prescription during the 12 months before conception and up to 1 day before the end of pregnancy.

In a sensitivity analysis, we excluded women with exposure to antiepileptic medication, antipsychotic medication, and insulin (ATC codes: N03A, N05A, and A10A).

### Maternal psychiatric illness

Information on psychiatric illness was obtained from the Danish Psychiatric Central Registry, which includes information on all admissions to psychiatric hospitals since 1970 as well as information on all psychiatric outpatient contacts since January 1995 [9]. From 1970 to 1993 psychiatric disorders were coded according to the ICD-8 and from January 1994 according to the ICD-10. Women were categorized as depressed during pregnancy if they had received a diagnosis of depression (ICD-10: F32–F33) any time in the interval from 6 months prior to conception up to 1 day before the end of the index pregnancy. History of severe mental disorder was defined as previous or ongoing diagnosis of bipolar disorder including mania or schizophrenia (ICD-8: 296.1–296.8, 298.1 and 295; ICD-10: F30–F31 and F20), and history of misuse was defined as previous or ongoing diagnosis of alcohol or drugs abuse (ICD-10: F10–F19).

The association between antidepressant use during pregnancy and spontaneous abortions was first valuated by comparing the risk of spontaneous abortion in pregnancies exposed to antidepressant medication with the risk in unexposed pregnancies regardless of a registry-based diagnosis of depressive disorder in the pregnant women, and thereafter by conducting the analyses stratified on registry-based diagnoses of depressive disorders during pregnancy.

We had no information on psychiatric diagnoses in women treated by a general practitioner or a private psychiatrist. As a consequence, for these women we had no comparison group of women with comparable depression but no use of antidepressants (discussed below).

### Covariates

Information on the following potential confounders was obtained from Statistics Denmark and subsequently coded as shown in parenthesis: maternal age at conception (years), cohabitation at time of conception (yes/no), income at time of conception (quartiles), and education level at time of conception (<10, 10–12, >12 years). From the Registry of Medicinal Product Statistics we obtained information on use of other drugs: Antipsychotic drugs (yes/no), antiepileptic drugs (yes/no), insulin (yes/no), and any reimbursed medication (yes/no); and from the Danish Psychiatric Central Registry we obtained information on psychiatric morbidity: severe mental disorder (yes/no), and drug abuse (yes/no).

### Statistics

Risk ratios (RR) for spontaneous abortion were estimated by using binomial regression with robust variance estimation to allow for correlation between pregnancy outcomes of each woman. RRs for spontaneous abortion were adjusted (aRR) for maternal age (age, age squared, and age to the power three, if the regression parameters could be estimated, and age in three categories otherwise), cohabitation, income (above or below the median), education, history of severe mental disorder, and history of drug abuse. The models were evaluated using directed acyclic graphs (not shown) [10]. To avoid unreliable results, RR analysis was only performed when at least 5 exposed events, i.e. spontaneous abortions or stillbirths, were observed. The RRs for spontaneous abortion were estimated not only for women exposed to any antidepressant but also for women exposed to individual or major subgroups of antidepressants (SSRI, TCA and ‘other’). No adjustments could be made in the latter analyses due to sparse data. In the analyses we excluded pregnancies resulting in induced abortions, i.e. results on spontaneous abortion are calculated from the numbers on spontaneous abortion, stillbirth and live birth. An induced abortion makes follow-up incomplete as the pregnancy could potentially have ended in a spontaneous abortion, but treating induced abortion as a competing risk in a time-to-event setup would result in the same conclusions as those obtained from the chosen strategy. However, we performed sensitivity analyses where the pregnancies were censored at the time of the induced abortion. Statistical analyses were performed using Stata 12 (StataCorp, Texas, USA).

### Ethics

The study was approved by the Danish Data Protection Agency. All analyses were performed on encrypted and anonymized data in a protected environment under Statistics Denmark, and the researchers had no access to the personal identifier (the CPR-number). Further, care was taken not to present results that might be identifiable. Under these conditions, no consent is needed from the participants under Danish law.

## Results

We identified 1,005,319 pregnancies of which 114,721 (11.4%) resulted in a spontaneous abortion. We found 22,061 pregnancies where the women used antidepressants during early pregnancy (“exposed cohort”), 1843 pregnancies in women with registry-based diagnosis of depression but no reported antidepressant use (“unexposed depression cohort”), and 981,415 pregnancies in women with no registry-based diagnosis of depression and no antidepressant use during pregnancy (“unexposed non-depression cohort”). Characteristics of the women are summarized in Table 1, and the outcomes of the pregnancies are summarized in Table 2. Both Table 1 and Table 2 are based on the whole cohort of women and their pregnancies. The following results are based the cohort where we excluded pregnancies for which exposure status of women were considered uncertain, as previously described. Table 3 shows aRRs according to exposure to antidepressants and to diagnosis of maternal depressive disorder. We found a slightly higher aRR for spontaneous abortion of 1.14 (95% CI 1.10–1.18) for women with overall antidepressant use when compared to women with no antidepressant use. However, we found no association when restricting the cohort to women with a registry-based diagnosis of depressive disorder (aRR 1.00; 95% CI 0.80–1.24). Among women with no registry-based diagnosis of depressive disorder (i.e. treated by a general practitioner or private psychiatrist), the aRR for spontaneous abortion was 1.17 (95% CI 1.13–1.22).

**Table 1:** Baseline Characteristics According to Diagnosis of Depression and Use of Antidepressants (AD).

	Depression & no AD use	Use of AD (+/−depression)	No depression & no AD use
All pregnancies (n)	1843	22061	981415
Maternal age (mean)	29.1 (sd = 6.3)	30.9 (sd = 5.9)	30.2 (sd = 5.5)
Maternal age, missing (n)	0 (0.0%)	0 (0.0%)	47 (0.0%)
Cohabitation (n)			
Yes	1014 (55.0%)	13579 (61.6%)	754610 (76.9%)
No	821 (44.5%)	8382 (38.0%)	211605 (21.6%)
Missing	8 (0.4%)	100 (0.5%)	15200 (1.5%)
Income (n)			
[0,20[ %	538 (29.2%)	4700 (21.3%)	194560 (19.8%)
[20–40[ %	518 (28.1%)	5535 (25.1%)	193749 (19.7%)
[40,60[ %	375 (20.3%)	4923 (22.3%)	194503 (19.8%)
[60–80[ %	225 (12.2%)	3790 (17.2%)	195785 (19.9%)
[80,100] %	185 (10.0%)	3111 (14.1%)	196505 (20.0%)
Missing	2 (0.1%)	2 (0.0%)	6313 (0.6%)
Education (n)			
<10 years	396 (21.5%)	4028 (18.3%)	105486 (10.7%)
10–12 years	777 (42.2%)	8369 (37.9%)	300635 (30.6%)
>12 years	625 (33.9%)	9171 (41.6%)	540257 (55.0%)
Missing	45 (2.4%)	493 (2.2%)	35037 (3.6%)
Co-medication (n)			
Antipsychotics	69 (3.7%)	1539 (7.0%)	1556 (0.2%)
Antiepileptics	26 (1.4%)	758 (3.4%)	3938 (0.4%)
Insuline	6 (0.3%)	188 (0.9%)	4465 (0.5%)
Any medication	1275 (69.2%)	22061 (100.0%)	607603 (61.9%)
Co-morbidity (n)			
History of severe mental disorder	76 (4.1%)	648 (2.9%)	2012 (0.2%)
History of drug abuse	136 (7.4%)	1073 (4.9%)	4395 (0.4%)
History of any psychiatric disorder	1843 (100.0%)	9997 (45.3%)	51613 (5.3%)

**Table 2:** Pregnancy Outcome According to Diagnosis of Depression and Use of Antidepressants (AD).

	Depression & no AD use	Use of AD (+/−depression)	No depression & no AD use
Pregnancy outcomes (n)	1858	22317	996787
Live birth	1044 (56.2%)	12730 (57.0%)	710779 (71.3%)
Stillbirth	5 (0.3%)	96 (0.4%)	3689 (0.4%)
Abortion	809 (43.5%)	9491 (42.5%)	282319 (28.3%)
divided acto.			
spontaneous abortion			
O02.0–O03.9	205 (11.0%)	2637 (11.8%)	111879 (11.2%)
induced abortion			
O04.0–O05.2, O05.5–O06.9	600 (32.3%)	6799 (30.5%)	167435 (16.8%)
O05.3–O05.4	4 (0.2%)	55 (0.2%)	3005 (0.3%)

**Table 3:** Stratified Analyses according to Diagnosis of Depression.

	Diagnosis of depression		No diagnosis of depression		Marginal	
	Exposed	Unexposed	Exposed	Unexposed	Exposed	Unexposed
Event	210	105	2427	110377	2637	110482
No event	1464	715	11362	708049	12826	708764
Risks^a^	0.125	0.128	0.176	0.135	0.171	0.135
RR	0.98 (0.78;1.23)		1.31 (1.26;1.35)		1.26 (1.22;1.31)	
aRR^b^	1.00 (0.80;1.24)		1.17 (1.13;1.22)		1.14 (1.10;1.18)	

a Probabilites of event.

b Adjusted for maternal age, cohabitation, income, education, history of severe mental disorder and drug abuse in case of the marginal analysis and the stratified analysis for no diagnosis of depression.

Adjusted for maternal age, cohabitation, education, and history of severe mental disorder in case of the stratified analysis for a diagnosis of depression.

**Figure 1:**
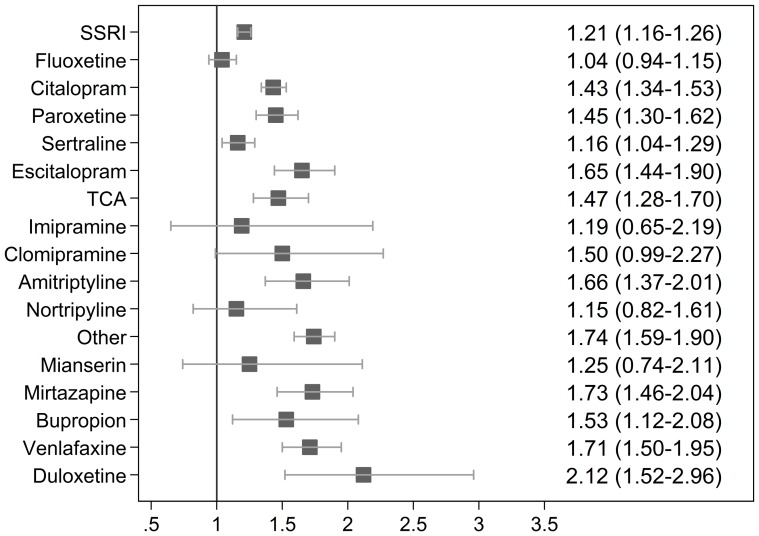
Unadjusted RR for spontaneous abortion after exposure to specific types of antidepressants compared to the unexposed cohort.

**Figure 2:**
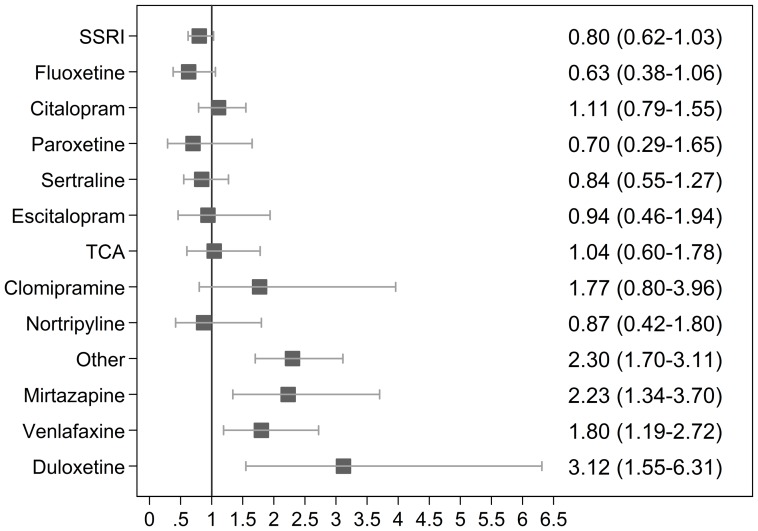
Unadjusted RR for spontaneous abortions after exposure to specific types of antidepressants among women with a hospital-based diagnosis of depression.

Figure 1 illustrates the (unadjusted) RR for spontaneous abortion after exposure to specific types of antidepressants compared to the unexposed cohort; Figure 2 illustrates the (unadjusted) RR for spontaneous abortion among women with a registry-based diagnosis of depression. Mirtazapine (RR 2.23; 95% CI 1.34–3.70), venlafaxine (RR 1.80; 95% CI 1.19–2.72), and duloxetine (RR 3.12; 95% CI 1.55–6.31) were associated with an increased risk of spontaneous abortion when restricting the analysis to women with a registry-based diagnosis of depressive disorder.

When excluding women exposed to antiepileptic drugs, antipsychotic drugs, and insulin we obtained similar results (results not shown). Notably, when restricting the cohort to women with a registry-based diagnosis of depressive disorder, there was no association between all antidepressant use and spontaneous abortions (aRR 0.98; 95% CI 0.77–1.23).

In sensitivity analyses, we found comparable results when women who redeemed a prescription of antidepressants 6 to 1 month before conception but not during pregnancy were categorized as exposed respectively unexposed (results not shown). In further sensitivity analyses, we defined a pregnancy as unexposed only if the pregnant woman had no redemption of antidepressant medication from 12 months prior to conception to the end of the index pregnancy. These analyses also showed comparable results (results not shown).

We found higher occurrence of induced abortions among women with a diagnosis of depression and/or use of antidepressants (Table 2). However, we found no sign of differential timing of these terminations within the exposure groups (results not shown).

## Discussion

Antidepressant use in pregnancy was associated with a slightly higher risk of spontaneous abortion overall, but no association was found when the analyses were restricted to women with an in- or outpatient hospital diagnosis of depressive disorder 6 months before conception or during pregnancy. No individual SSRI was associated with spontaneous abortions in this group but specific antidepressants with effects on the serotonin and norepinephrine systems (mirtazapine, venlafaxine, and duloxetine) were associated with spontaneous abortions. This may, however, reflect residual confounding by depression severity.

In a meta-analysis from 2005, Hemels et al. found that SSRIs and dual action antidepressants (including venlafaxine) were associated with spontaneous abortions [3]. The included studies were heterogenic in respect to exposure and outcome definitions and none of the studies estimated the rate of spontaneous abortions among unexposed, depressed women. In a recent register-based case-control study, Nakhai-Pour et al. found a 68% increase in the risk of spontaneous abortions (aRR 1.68; 95% CI 1.38–2.06) after exposure to SSRIs [4] comparable to the estimate of Hemels et al. The study adjusted for history of depression, anxiety and use of psychiatric services, but did not compare with unexposed women with depression. The overall rates of clinically recognized spontaneous abortions in the earlier papers were comparable to what we found.

In analyses of individual drugs, we found that mirtazapine, venlafaxine, and duloxetine were associated with spontaneous abortions also among women with a registry-based diagnosis of depression. In this group, we found no associations between TCA or individual SSRIs and spontaneous abortions. The result is corroborated by Pastuszak et al. that found the same risk of spontaneous abortions associated with fluoxetine and TCA [11]. Nakhai-Pour et al. found the strongest association for venlafaxine but, unlike us, also for paroxetine [4]. Hemels et al. did not have sufficient sample size to estimate the risk associated with individual SSRIs [3].

Maternal depression and associated life-style factors are important when interpreting both the present and previous results. Nakano et al. found that depression, attribution and social support were predictors of pregnancy outcomes in women with recurrent spontaneous abortions [12]. Our results of the analyses on women with a registry-based diagnosis of depression suggest, that confounding by indication could, at least partly, explain the association between antidepressants and spontaneous abortions. Concerning the estimates of the individual types of antidepressants, the underlying disease severity, nature, or associated life-style factors may vary substantially between women treated with the different types of antidepressants. Mirtazapine, venlafaxine, or duloxetine are not the first or even second choice during pregnancy, and women treated with one of these antidepressants may thus suffer from a more severe or treatment-resistant disorder. However, the drugs have different pharmacokinetic and –dynamic properties from the other antidepressants (e.g. SSRIs), and the associations may indicate an abortogenic effect associated with changes in the norepinephrine system but larger studies are needed.

### Limitations

The study attempts to disentangle the effects of maternal depression and antidepressant exposure by using information from nationwide registries. We defined women as depressed if they in the Danish Psychiatric Central Registry had received an ICD-10 code of depression 6 months prior to conception or during pregnancy. Women coded as depressed in the Danish National Hospital Registry only, and not in the Danish Psychiatric Central Registry, were not classified as depressed. Conversely, women with a less severe degree of depression treated by a general practitioner or with no contact during the exposure period were not coded as depressed. Approximately 80% of the women who used antidepressants during pregnancy were treated in the primary care system only and we had consequently no information on the underlying psychiatric disorder.

We adjusted for available potential confounders and attempted to take the underlying disease (confounding by indication) into account in the design of the study. We recognize that residual and uncontrolled confounding may still be a problem in this study as in other studies without random allocation of exposure. Furthermore, we had no information on important potential confounders, including smoking, alcohol intake, and diet, and, importantly, the group of women with non-medicated depressive disorder is not a perfect comparison group, as they apparently differ in other aspects than antidepressant use.

Some pregnancies that end in spontaneous abortions, in particular very early in pregnancy, are not recorded in the hospital registry. The symptoms may be interpreted as a late menstrual period or treated outside the hospital system. In a system with free, universal health care the number of spontaneous abortions treated outside the hospital system will decrease dramatically with increased gestational age. If the propensity for hospital contact or admission is associated with the exposure, it will lead to information bias [13], although we found no sign of this particular type of bias when investigating the timing of spontaneous abortions. Women who used antidepressants more often terminated their pregnancy but taking this competing risk into consideration did not change the estimates much indicating that the outcome was not related to the risk of spontaneous abortion.

The study had very limited loss to follow-up owing to the completeness of the population-based registries and limited emigration. Selection bias may, however, still occur if a covariate in the models was associated with an underlying cause of the exposure and was itself a consequence of the outcome. Our directed acyclic graphs did not, however, suggest such collider bias even in the analyses stratified on registry-based diagnosis of maternal depression.

There is a risk of exposure misclassification in the use of redeemed prescriptions to define exposure. For drug use overall, the risk seems to depend on the type of medication, i.e. the correlation between the time of redemption and actual use will be low for migraine medication and high for antibiotics [14]. For the women defined as exposed to antidepressants, this misclassification is likely to bias results toward the null [14]. Conversely, for women defined as depressed with no antidepressant use, a potential drug exposure misclassification (e.g. if the women received treatment during an inpatient admission and redeemed no prescription to antidepressants after discharge) will lead to a bias away from the null in the main analyses comparing women with antidepressant use with the “non-medicated depression cohort”. In sensitivity analyses with “washout periods” up to 12 months we found no change suggesting that misclassification is not a likely cause of the main findings.

## Conclusion

We identified a slightly increased risk of spontaneous abortion associated with the use of antidepressants during pregnancy that may be related to the underlying maternal depression or factors related to the disorder. Use of SSRIs was not associated with spontaneous abortions among women with a registry-based diagnosis of depression; in contrast, in unadjusted analyses the risk was increased after use of specific antidepressants with effects on both the serotonin and norepinephrine systems, which warrants further studies.
